# Потеря волос на фоне лечения ожирения: современное состояние проблемы и потенциальные решения

**DOI:** 10.14341/probl13817

**Published:** 2026-09-08

**Authors:** Е. А. Трошина, Л. А. Суплотова, Ф. Х. Дзгоева, Л. А. Руяткина, А. Г. Гаджигороева, О. А. Громова, Ю. Ю. Романова

**Affiliations:** Национальный медицинский центр эндокринологии им. академика И.И. Дедова Россия; Endocrinology Research Centre Russian Federation; Тюменский государственный медицинский университет Россия; Tyumen State Medical University Russian Federation; Новосибирский государственный медицинский университет Россия; Novosibirsk State Medical University Russian Federation; Российский университет дружбы народов им. Патриса Лумумбы; Московский научно-практический Центр дерматовенерологии и косметологии Россия; Patrice Lumumba Peoples’ Friendship University; Moscow Scientific and Practical Center of Dermatovenereology and Cosmetology Russian Federation; Федеральный исследовательский центр «Информатика и управление» Российской Академии Наук; Ивановский государственный медицинский университет Россия; Federal Research Center «Informatics and Control» of the Russian Academy of Sciences; Ivanovo State Medical University Russian Federation; Российский университет дружбы народов им. Патриса Лумумбы Россия; Patrice Lumumba Peoples’ Friendship University Russian Federation

**Keywords:** ожирение, избыточная масса тела, выпадение волос, алопеция, телогеновая алопеция, андрогенетическая алопеция, препараты инкретинового ряда, агонисты рецептора глюкагоноподобного пептида-1, биотин, витамин D, микронутриенты, obesity, overweight, hair loss, alopecia, telogen effluvium, androgenetic alopecia, incretin-based therapies, glucagon-like peptide-1 receptor agonists, biotin, vitamin D, micronutrients

## Abstract

Ожирение и избыточная масса тела являются значимыми медико-социальными проблемами, а современные подходы к их лечению, включая гипокалорийные диеты, бариатрические вмешательства и фармакотерапию, в том числе препаратами инкретинового ряда (например, агонистами рецептора глюкагоноподобного пептида-1), могут сопровождаться развитием диффузного выпадения волос. Настоящая статья подготовлена по результатам обсуждения на совете экспертов и систематизирует современные данные о распространенности и клинико-морфологических вариантах алопеции на фоне снижения массы тела. Рассмотрены основные патогенетические механизмы, включая метаболический стресс, белково-энергетическую недостаточность и дефицит микронутриентов. Отдельное внимание уделено роли биотина и витамина D в поддержании функций волосяного фолликула, а также группам риска развития алопеции у пациентов, получающих лечение по поводу ожирения. Представлены подходы к профилактике, мониторингу и комплексному ведению таких пациентов с учетом необходимости сохранения приверженности основной терапии.

В статье систематизированы современные данные о распространенности и клинико-морфологических вариантах алопеции на фоне снижения массы тела, рассмотрены основные патогенетические механизмы, включая метаболический стресс, белково-энергетическую недостаточность и дефицит микронутриентов. Отдельное внимание уделено роли биотина и витамина D в поддержании функций волосяного фолликула, а также группам риска развития алопеции у пациентов, получающих лечение по поводу ожирения. Представлены подходы к профилактике, мониторингу и комплексному ведению таких пациентов с учетом необходимости сохранения приверженности основной терапии.

## Abstract (en)

Obesity and overweight are significant medical and social problems, and current approaches to their treatment, including hypocaloric diets, bariatric interventions, and pharmacotherapy, including incretin drugs (e.g., glucagon-like peptide-1 receptor agonists), can be accompanied by the development of diffuse hair loss. This article, prepared following a discussion at an expert council, systematizes current data on the prevalence and clinical and morphological variants of alopecia associated with weight loss. The main pathogenic mechanisms are discussed, including metabolic stress, protein-energy malnutrition, and micronutrient deficiencies.

Special attention is paid to the role of biotin and vitamin D in maintaining hair follicle function, as well as to risk groups for alopecia in patients undergoing treatment for obesity. Approaches to the prevention, monitoring, and comprehensive management of such patients are presented, taking into account the need to maintain adherence to the main therapy.

This article systematizes current data on the prevalence and clinical and morphological variants of alopecia associated with weight loss. It examines the main pathogenic mechanisms, including metabolic stress, protein-energy malnutrition, and micronutrient deficiencies. Special attention is paid to the role of biotin and vitamin D in maintaining hair follicle function, as well as to risk groups for alopecia in patients undergoing treatment for obesity. Approaches to prevention, monitoring, and comprehensive management of these patients are presented, taking into account the need to maintain adherence to the primary therapy.

## ВВЕДЕНИЕ

Ожирение и избыточная масса тела относятся к числу наиболее значимых медико-социальных проблем в Российской Федерации (РФ). По данным статистического сборника Росстата «Здравоохранение в России — 2025», в 2024 г. в стране было зарегистрировано около 3 млн пациентов с ожирением; впервые установленный диагноз ожирения был поставлен 542,2 тыс. человек, а показатель общей зарегистрированной заболеваемости составил 2055,1 на 100 000 населения. По данным выборочных обследований состояния здоровья и питания населения Росстата, избыточная масса тела и ожирение сохраняют высокую распространенность во всех федеральных округах, что подтверждает значительную популяционную нагрузку данного заболевания [1].

Согласно клиническим рекомендациям Минздрава России «Ожирение» (2024), снижение массы тела показано всем пациентам с ожирением, а также пациентам с избыточной массой тела при наличии факторов риска или ассоциированных заболеваний; целевым ориентиром является уменьшение массы тела на 5–10% в течение 3–6 месяцев с удержанием результата не менее года. В рекомендациях подчеркивается необходимость комплексного подхода, включающего модификацию образа жизни, диетотерапию, фармакотерапию и, у отдельных пациентов, бариатрическое лечение [2–5]. Ожирение рассматривается как состояние избыточного питания, однако парадоксальным образом может быть ассоциировано с дефицитами ряда микронутриентов [6]. Наиболее подробно изучена обеспеченность витамином D: согласно метаанализу 23 исследований, его распространенность на 35% выше у пациентов с ожирением по сравнению с лицами с нормальной массой тела [7].

Выпадение волос и ухудшение их качества могут наблюдаться на фоне различных методов снижения массы тела, включая гипокалорийные диеты, бариатрические вмешательства и фармакотерапию препаратами инкретинового ряда, в том числе агонистами рецептора глюкагоноподобного пептида-1 (аГПП-1). В обновленном систематическом обзоре и метаанализе 2025 г. частота выпадения волос после метаболической и бариатрической хирургии составила 47% (41 исследование, 7044 пациента). Более ранний метаанализ 2021 г. показал интегральную оценку частоты в 57%, а также сопоставимо высокую частоту выпадения волос после рукавной гастрэктомии и желудочного шунтирования по Roux-en-Y [8][9].

Эпидемиологические данные свидетельствуют о тесной связи диффузного выпадения волос в процессе снижения массы тела с ее скоростью и выраженностью. В ретроспективном исследовании телогенового выпадения волос, ассоциированного с похудением, средняя потеря массы тела составила 15,21%, средняя скорость снижения массы — 3,54 кг/месяц, а клинические проявления выпадения волос в среднем возникали через 1,12 месяца от начала похудения. Эти данные согласуются с представлением о телогеновом выпадении волос как о проявлении метаболического и нутритивного стресса [10].

При применении арГПП-1 (семаглутид, лираглутид и др.) частота сообщений о случаях алопеции в пострегистрационных исследованиях достигает 10% и более, однако имеющаяся доказательная база преимущественно основана на наблюдательных данных [11–13]. В исследовании, опубликованном в журнале Skin Appendage Disorders (2026), выпадение волос после начала терапии арГПП-1 отметили 70,4% опрошенных пациентов; чаще это регистрировалось у женщин и у лиц с более выраженным снижением массы тела [12]. Частота назначений арГПП-1 неуклонно растет в последние годы, в связи с чем следует ожидать увеличение обращаемости к дерматологам по поводу выпадения волос на фоне их приема. Эстетические последствия могут оказывать значительное влияние на приверженность к терапии ожирения: до 39,7% пациентов с выпадением волос на фоне приема арГПП-1 указывают его в качестве причины прекращения лечения [12]. Сообщается об ассоциации выпадения волос на фоне арГПП-1 с темпами снижения массы тела, а также с конкретно используемым препаратом (чаще при приеме тирзепатида), дозами и продолжительностью использования препаратов [14]. В крупном когортном анализе применение арГПП-1 ассоциировалось с увеличением риска развития андрогенетической алопеции через 6 мес терапии на 62% и телогеновой алопеции через 12 мес на 76%. Подобные диффузные формы выпадения волос описаны и у пациентов, соблюдающих выраженно гипокалорийные диеты или перенесших бариатрические операции, что связывают с острым метаболическим стрессом и дефицитом нутриентов [12].

Анализ доступных данных показывает, что наиболее частыми клинико-морфологическими вариантами выпадения волос на фоне терапии арГПП-1 являются телогеновое выпадение и андрогенетическая алопеция (АГА); при этом в большинстве исследований отсутствует стандартизированная дерматологическая верификация диагноза. Обзорные публикации последних лет указывают, что причинно-следственная связь между терапией арГПП-1 и выпадением волос пока окончательно не доказана, однако имеющийся массив данных позволяет рассматривать алопецию как клинически значимую потенциальную нежелательную реакцию, требующую настороженности и активного мониторинга [5][16–19]. Метаболические факторы (инсулинорезистентность, ожирение, метаболический синдром) участвуют в патогенезе АГА — хронической прогрессирующей потери волос в андроген-чувствительных зонах скальпа. Согласно метаанализу данных 2026 г., наличие у пациентов ожирения ассоциировано с прогрессированием АГА (ОШ 2,31; 95% ДИ 1,01–5,29). Инсулинорезистентность и высокий уровень инсулина натощак определены как факторы, повышающие риск развития АГА [20]. Хотя терапия ожирения может сопровождаться усилением потери волос, сопутствующие изменения метаболического профиля потенциально могут положительно сказываться на течении АГА [14].

## ВОЗМОЖНЫЕ ПАТОГЕНЕТИЧЕСКИЕ МЕХАНИЗМЫ СИНДРОМА АЛОПЕЦИИ, АССОЦИИРОВАННОЙ СО СНИЖЕНИЕМ МАССЫ ТЕЛА, И РОЛЬ БИОТИНА В ИХ КОРРЕКЦИИ

Наиболее вероятными клинико-патогенетическими механизмами выпадения волос на фоне лечения ожирения являются ускоренный перевод волосяных фолликулов в телогеновую фазу под влиянием дефицита белка и микронутриентов (железо, цинк, витамин D, биотин и др.), а также влияние исходных гормональных и воспалительно-метаболических нарушений у пациентов с ожирением и инсулинорезистентностью [4][8][16].

Биотин (витамин B7) является водорастворимым витамином комплекса B и играет важную роль в метаболизме клеток волосяного фолликула и регуляции роста волос [21]. При этом молекула биотина имеет 8 стереоизомеров, но единственным биологически активным является D-биотин. Биотин выступает кофактором ряда карбоксилаз, обеспечивающих процессы глюконеогенеза, обмена аминокислот и синтеза жирных кислот, а также участвует в посттрансляционной модификации гистоновых белков, влияя на экспрессию генов, связанных с ростом волос [22]. Согласно метагеномному анализу у 1545 субъектов когортного исследования MetaCardis, тяжелое ожирение (ИМТ≥35 кг/м²) сопровождается дефицитом кишечных бактерий-продуцентов и транспортеров биотина, что ассоциировано с худшими метаболическими и воспалительными параметрами пациентов (ИМТ, окружность талии, уровень глюкозы натощак, HbA1c, С-пептид, триглицериды и др.). При оценке уровня биотина в сыворотке крови в подгруппе участников когорты у 78,09% пациентов с тяжелым ожирением установлен субоптимальный или дефицитный уровень биотина — против 19,65% у лиц с нормальной массой тела [23].

Экспериментальные данные указывают на способность биотина усиливать экспрессию генов, способствующих росту волос, повышать экспрессию кератинов и модулировать активность ростовых факторов и сигнальных путей, значимых для функции волосяного фолликула [24].

Указанные биологические эффекты лежат в основе применения биотина в составе комплексной терапии диффузной алопеции в дерматологической и трихологической практике. Вместе с тем данные рандомизированных контролируемых исследований эффективности различных доз биотина при выпадении волос остаются ограниченными и неоднородными [25][26].

На территории РФ зарегистрирован лекарственный препарат D-биотина в терапевтической дозировке, для которого проведено регистрационное клиническое исследование №МА/1217-1 «Двойное слепое плацебо-контролируемое многоцентровое рандомизированное исследование эффективности и безопасности препарата биотин, таблетки, 10 мг (Фармацевтический завод “ПОЛЬФАРМА” АО, Польша) в терапии пациентов с выпадением волос и снижением густоты волос» — «Биотиналь В7», 10 мг, ЛП-№(002640)-(РГ-RU). Биотиналь В7 может быть рекомендован для применения в данной клинической ситуации курсами по 3 месяца на основании утвержденных регистрационных документов. Следует отметить, что практика назначения лекарственных препаратов D-биотина в дозировке 10 мг в контексте лечения синдрома алопеции, ассоциированного со снижением массы тела при ожирении, требует дальнейшей систематизации и клинико-фармакологической оценки [27][28].

## ВОЗМОЖНЫЕ ПАТОГЕНЕТИЧЕСКИЕ МЕХАНИЗМЫ СИНДРОМА АЛОПЕЦИИ, АССОЦИИРОВАННОЙ СО СНИЖЕНИЕМ МАССЫ ТЕЛА, И РОЛЬ ВИТАМИНА D В ИХ КОРРЕКЦИИ

Значительное ограничение калорийности и быстрое снижение массы тела являются хорошо известными факторами, провоцирующими холестаз, гиперсатурацию желчи и желчнокаменную болезнь [29]. С учетом гипокинезии желчевыводящих путей, снижения поступления желчи в двенадцатиперстную кишку и возможных нарушений всасывания жиров, возникающих при быстром снижении массы тела, в т.ч. на фоне терапии препаратами инкретинового ряда и после бариатрических вмешательств, у пациентов может развиваться и прогрессировать дефицит витамина D.

Дефицит витамина D в настоящее время рассматривается как значимый патогенетический фактор телогеновой алопеции, опосредующий нарушение регуляции цикла роста волос и функциональной активности волосяного фолликула. Через рецептор витамина D, экспрессируемый в кератиноцитах и клетках дермального сосочка, активные метаболиты витамина D участвуют в индукции и поддержании анагеновой фазы, дифференцировке клеток матрикса и синтезе структурных белков волоса. При снижении уровня 25(OH)D отмечается дискоординация переходов анаген–катаген–телоген, что клинически может проявляться диффузным телогеновым выпадением волос.

Наряду с этим дефицит витамина D ассоциирован с изменением локального иммунного гомеостаза и усилением провоспалительных реакций в перифолликулярной зоне. Это может способствовать преждевременному переходу фолликулов в состояние покоя и усугублению телогеновой алопеции [30–32]. Результаты клинических наблюдений показывают, что у пациентов с телогеновой алопецией уровни 25(OH)D, как правило, ниже по сравнению со здоровыми лицами, а коррекция гиповитаминоза D сопровождается снижением интенсивности выпадения волос и улучшением дерматоскопических показателей [33].

## ГРУППЫ РИСКА РАЗВИТИЯ АЛОПЕЦИИ НА ФОНЕ ЛЕЧЕНИЯ ОЖИРЕНИЯ

К предикторам избыточного выпадения волос у пациентов, начинающих терапию ожирения и избыточной массы тела, целесообразно относить следующие факторы:

Выделение таких групп риска позволяет заранее планировать мониторинг и профилактические мероприятия, включая лабораторную оценку дефицитных состояний и дерматологическое наблюдение.

## ТАКТИКА ВЕДЕНИЯ И ПРОФИЛАКТИКА

Рекомендованная тактика ведения пациентов с ожирением и избыточной массой тела в контексте риска выпадения волос может включать несколько ключевых положений. Пациентов, начинающих терапию ожирения, в том числе при назначении арГПП-1 или комбинированных препаратов инкретинового ряда, необходимо информировать о возможном риске усиления выпадения волос для поддержания приверженности лечению. До начала терапии и через 3 мес после ее старта целесообразна оценка нутритивного статуса с определением уровней ферритина, сывороточного железа, цинка, 25(OH)D, а при наличии технической возможности — биотина в плазме крови; в тяжелых случаях алопеции — определение активности биотинидазы в крови.

Важными компонентами профилактики и коррекции являются устранение дефицита витамина D3 с достижением целевой концентрации 25(OH)D более 30 нг/мл (лечение следует проводить согласно проекту клинических рекомендаций РАЭ «Дефицит витамина D») [34] и обеспечение адекватного поступления белка не менее 1,2–1,5 г/кг идеальной массы тела.

С учетом гипокинезии желчевыводящих путей, снижения поступления желчи в двенадцатиперстную кишку и возможных нарушений всасывания жиров обосновано использование водорастворимых мицеллярных форм витамина D, всасывание которых в меньшей степени зависит от секреции желчи [35–37]. У пациентов с клинически значимым выпадением волос на фоне фармакологического и нефармакологического лечения ожирения, особенно при подтвержденном или предполагаемом дефиците биотина, может быть рекомендован прием препарата Биотиналь В7 курсами по 3 мес [38][39].

## ЗАКЛЮЧЕНИЕ

Диффузное выпадение волос — прежде всего телогеновая алопеция и андрогенетическая алопеция — является частым и клинически значимым неблагоприятным явлением современных методов лечения ожирения, включая фармакотерапию, бариатрическую хирургию и интенсивные диетические ограничения. Патогенез алопеции в этих ситуациях носит многофакторный характер; ключевую роль играют острый метаболический стресс, дефицит белка и эссенциальных микронутриентов, а также возможное прямое или опосредованное влияние препаратов инкретинового ряда на цикл волосяного фолликула. Жалобы на выпадение волос не должны автоматически приводить к отмене фармакотерапии ожирения без оценки соотношения ожидаемой пользы и потенциального риска.

Наиболее рациональной представляется междисциплинарная тактика ведения пациентов с участием эндокринолога, дерматолога или трихолога, а при необходимости — диетолога. В процессе фармакотерапии ожирения рекомендуется систематически опрашивать пациентов о нарастающем выпадении волос, изменении их толщины, появлении очагов поредения, а также о влиянии этих симптомов на качество жизни и приверженность лечению. При появлении соответствующих жалоб целесообразно проводить клиническую оценку возможных причин, включая темпы снижения массы тела, характер питания, уровень белкового обеспечения, сопутствующие заболевания, стрессовые факторы, перенесенные инфекции, и выполнять лабораторный скрининг дефицитных состояний по клиническим показаниям.

Одновременная коррекция дефицита витамина D3 водорастворимой мицеллярной формой витамина D должна рассматриваться как обязательный компонент комплексной терапии. Обеспечение адекватного поступления белка является ключевым элементом профилактики и коррекции телогеновой алопеции у пациентов, снижающих массу тела. Учитывая биологическую роль биотина в механизмах обмена веществ в волосяных фолликулах и роста волос, а также положительный клинический опыт применения при алопеции, назначение лекарственных препаратов D-биотина может быть рекомендовано пациентам с алопецией на фоне лечения ожирения на протяжении всего периода активного снижения массы тела.

## ДОПОЛНИТЕЛЬНАЯ ИНФОРМАЦИЯ

Источники финансирования. Работа выполнена по инициативе авторов без привлечения финансирования.

Конфликт интересов. Авторы декларируют отсутствие явных и потенциальных конфликтов интересов, связанных с содержанием настоящей статьи.

Участие авторов. Все авторы одобрили финальную версию статьи перед публикацией, выразили согласие нести ответственность за все аспекты работы, подразумевающую надлежащее изучение и решение вопросов, связанных с точностью или добросовестностью любой части работы.
